# Supplementary figures and images for: Interkingdom interactions shape the fungal microbiome of mosquitoes
Source: Anim Microbiome. 2024 Mar 7;6:11. doi: 10.1186/s42523-024-00298-4 (PMC10921588; doi:10.1186/s42523-024-00298-4)

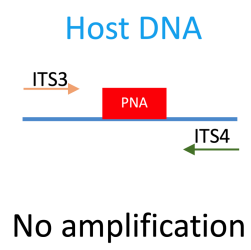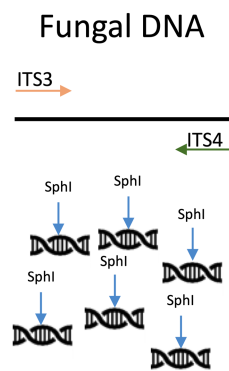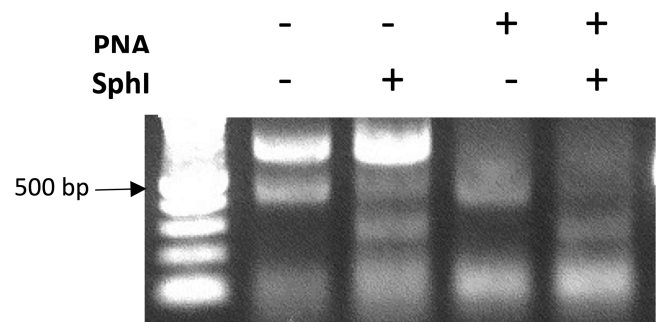

Supplement: Supplementary file 1 — Additional file1: PNA blocker PCR : (Left) Schematic representation of the PNA blocking the PCR amplifying the host ITS. The amplicon was digested with SphI, which specifically cuts the fungal ITS. (Right) Agarose gel showing the PCR products done with Ae. aegypti laboratory samples in presence or absence of PNA blocker. The PCR product was digested with SphI. [file 42523_2024_298_MOESM1_ESM.pdf]

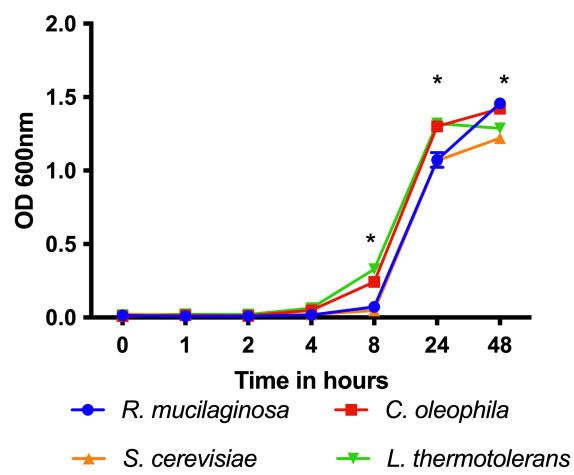

Supplement: Supplementary file 2 — Additional file2: In vitro growth analysis of fungi. The fungal isolates were grown in YPD medium at 28 C for 48 hrs and OD600 was recorded at indicated time points. The experiment was repeated twice each with 5 replicates. The data were analysed by two-way ANOVA with Tukey’s multiple comparision test. The assay was done twice each in 5 replicates. [file 42523_2024_298_MOESM2_ESM.pdf]

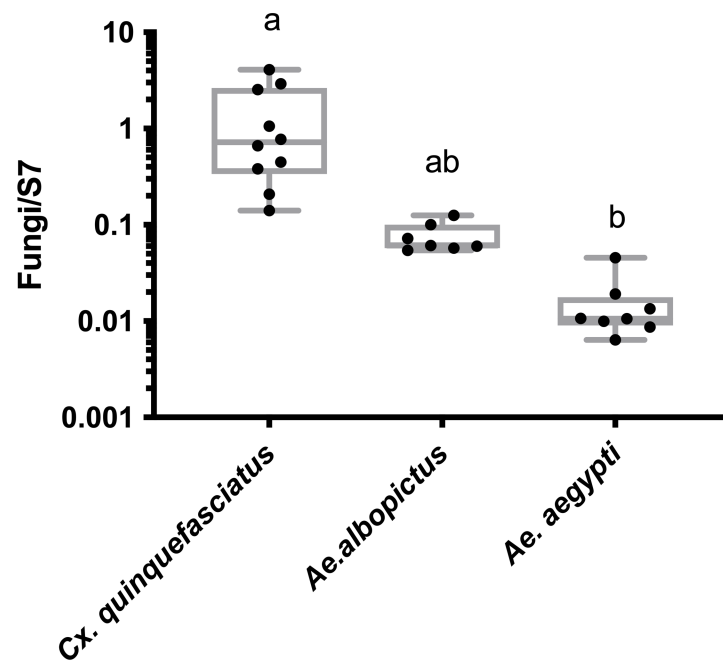

Supplement: Supplementary file 3 — Additional file3: Total fungal abundance. The fungal load in the laboratory reared mosquitoes is analysed by qPCR using primer specific 18S rRNA gene and host endogenous gene S7 and Actin were used as control. The Ct values were normalized to host genes are represented in the graph. The data were analysed by one-way ANOVA with Dunn’s multiple comparision test. The P<0.05 considered significant. [file 42523_2024_298_MOESM3_ESM.pdf]

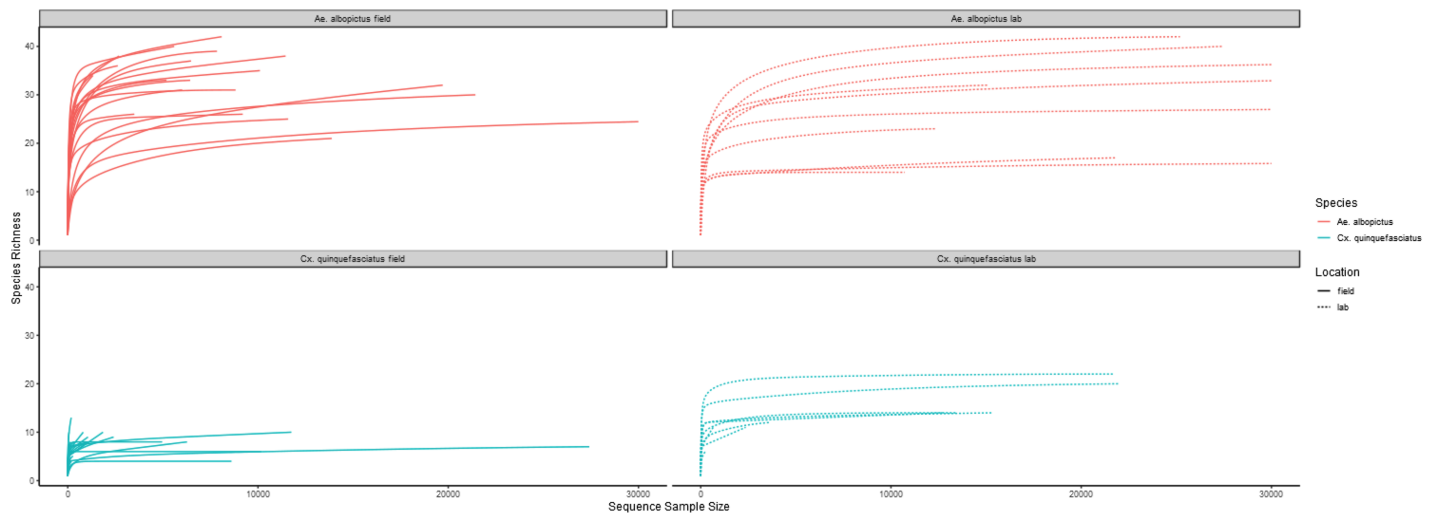

Supplement: Supplementary file 4 — Additional file4: Rarefaction curve: Alpha diversity species richness at intervals between 0 and 30,000 reads in each sample from different groups lab and field samples in Ae. albopictus and Cx. quinquesfasciatus. [file 42523_2024_298_MOESM4_ESM.pdf]

**A**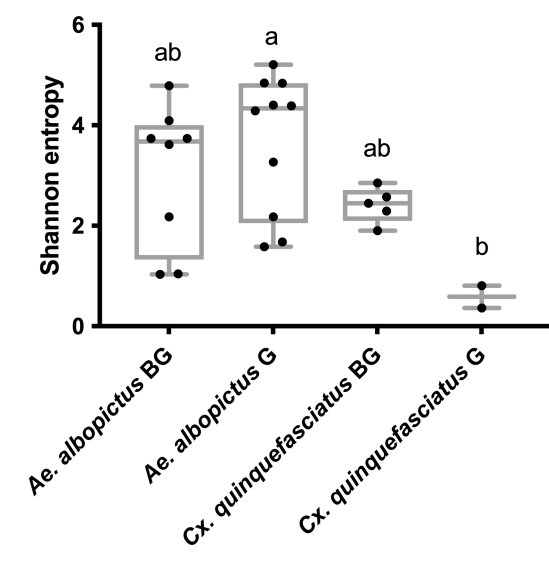**B**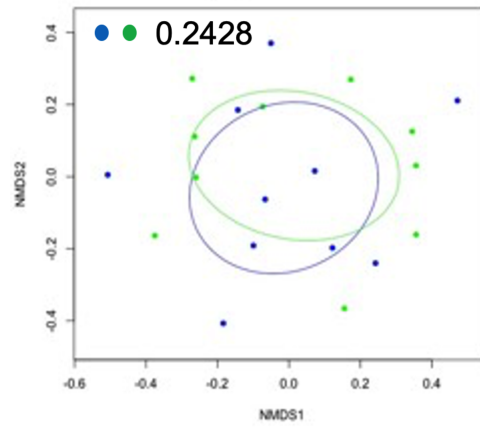**C**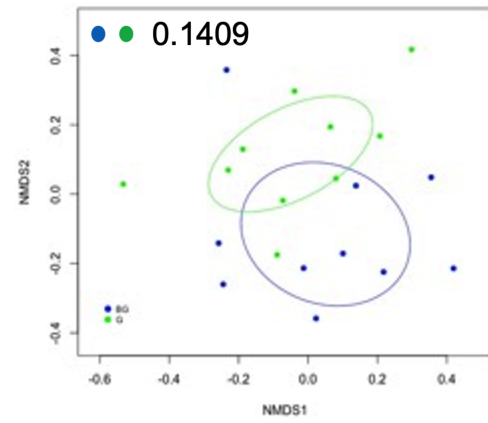

Supplement: Supplementary file 5 — Additional file5: Abundance and diversity of fungal microbiome field samples. (A) Alpha diversity analysis of fungal communities in Ae. albopictus and Cx. quinquefasciatus samples collected using gravid (G) and BG sentinel traps. The statistical significance was determined by one-way ANOVA with Tukey’s multiple comparison test. The P<0.05 considered significant. The diversity of communities in the G and BG samples of Ae. albopictus (B) and Cx. quinquefasciatus (C) were analysed by Bray-Curtis metric. [file 42523_2024_298_MOESM5_ESM.pdf]

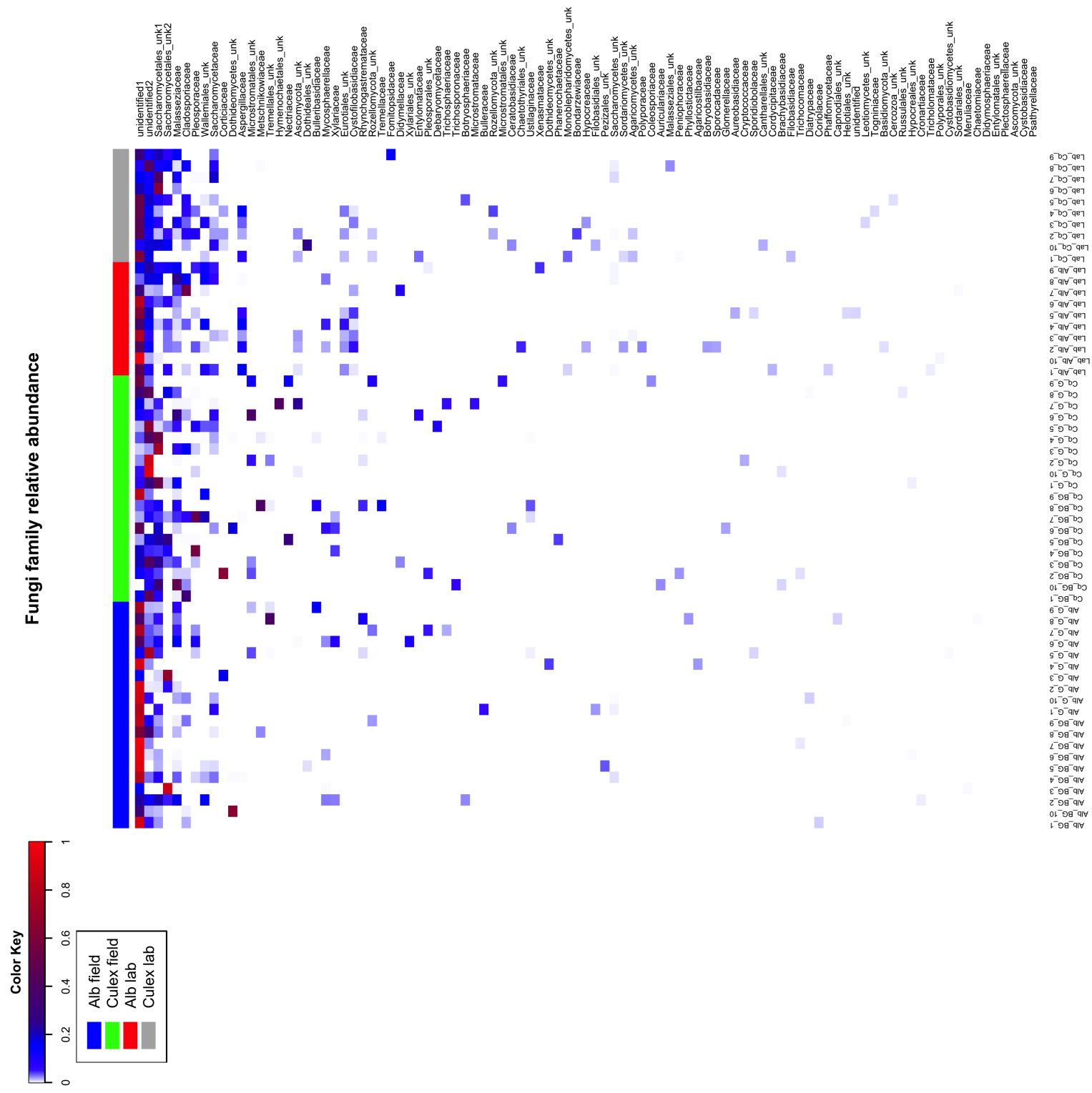

Supplement: Supplementary file 6 — Additional file6: Beta diversity analysis:The detailed view of the comparison of abundance at family level between Ae. albopictus and Cx. quinquefasciatus field and laboratory samples. [file 42523_2024_298_MOESM6_ESM.pdf]
